# Validation of loci and genes associated with fertility in Holstein cows using gene-set enrichment analysis-SNP and genotype-by-sequencing

**DOI:** 10.1186/s12864-025-11364-9

**Published:** 2025-02-21

**Authors:** Jennifer N. Kiser, Christopher M. Seabury, Mahesh Neupane, Joao G. N. Moraes, Allison L. Herrick, Joseph Dalton, Gregory W. Burns, Thomas E. Spencer, Holly L. Neibergs

**Affiliations:** 1 Department of Animal Sciences, Washington State University, Pullman, WA USA; 2 Department of Veterinary Pathobiology, College of Veterinary Medicine, Texas A&M University, College Station, TX USA; 3 Animal Genomics and Improvement Laboratory, United States Department of Agriculture, Beltsville, MD USA; 4 Department of Animal and Food Sciences, Oklahoma State University, Stillwater, OK USA; 5 Department of Animal, Veterinary and Food Sciences, University of Idaho, Caldwell, ID USA; 6 College of Agriculture and Natural Resources, Michigan State University, East Lansing, MI USA; 7 Division of Animal Sciences, University of Missouri, Columbia, MO USA

**Keywords:** Fertility, GBS, GSEA-SNP, Holstein cattle, Uterine receptivity

## Abstract

**Background:**

The financial strain fertility issues cause the dairy cattle industry is substantial, with over $7 billion in lost revenue accrued annually due to a relatively low cow conception rate (CCR; 30–43%) for US dairy cows. While CCR has been improving through genomic selection, identification of causal mutations would help improve the rate of genetic progress with genomic selection and provide a better understanding of infertility. The objectives of this study were to: (1) identify genes and gene-sets associated with CCR to the first breeding (CCR1) and the number of breedings required to conceive (TBRD) in Holstein cows and (2) identify putative functional variants associated with CCR1 and TBRD through a custom genotype-by-sequencing array. The study consisted of 1,032 cows (494 pregnant to first breeding, 472 pregnant to subsequent [2–20] services, and 66 that never conceived). Cows were artificially inseminated, and pregnancy was determined 35d later by rectal palpation of uterine contents. Gene-set enrichment analyses with SNP data (GSEA-SNP) were conducted for CCR1 and TBRD with a normalized enrichment score (NES) ≥ 3.0 required for significance. Leading edge genes (LEG) and positional candidate genes from this and 26 additional studies were used to validate 100 loci associated (*P* < 1 × 10^− 5^) with cow fertility using a custom sequencing genotyping array of putative functional variants (exons, promoters, splice sites, and conserved regions).

**Results:**

GSEA-SNP identified 95 gene-sets (1,473 LEG) enriched for CCR1 and 67 gene sets enriched (1,438 LEG) for TBRD (NES ≥ 3). Thirty-four gene-sets were shared between CCR1 and TBRD along with 788 LEG. The association analysis for TBRD identified three loci: BTA1 at 83 Mb, BTA1 at 145 Mb, and BTA 20 at 46 Mb (*P* < 1 × 10^− 5^). The loci associated with TBRD contained candidate genes with functions relating to implantation and uterine receptivity. No loci were associated with CCR1, however a single locus on BTA1 at 146 Mb trended toward significance with an FDR of 0.04.

**Conclusions:**

The validation of three loci associated with CCR and TBRD in Holsteins can be used to improve fertility through genomic selection and provide insight into understanding infertility.

**Supplementary Information:**

The online version contains supplementary material available at 10.1186/s12864-025-11364-9.

## Background

Revenues from dairy cattle products account for roughly 21.3% ($41.8 billion) of all animal cash receipts within the United States [1]. Worldwide, global revenue attributed to milk is expected to amount to $350 billion in 2024 and continue to grow in subsequent years [2]. Prior to lactation and the generation of dairy products, cattle must first conceive and maintain the pregnancy through the normal gestation period, making fertility critical to dairy profitability.

The dairy industry has struggled with fertility for decades, with the average cow conception rate (CCR) ranging between 30–43% [3–4]. Previous studies have estimated the average economic cost of pregnancy loss from breeding to day 60 of gestation in dairy cows to be $2,333 [5]. This cost is dependent on several factors including length of pregnancy, increased chance of culling, and the cost of rebreeding. To put this on a national scale, if 35% of the 9.38 million U.S. cows [6] lost a single pregnancy it would result in a cost of greater than $7.6 billion dollars to the dairy industry. This illustrates the importance of selecting cattle that can conceive and maintain pregnancies.

Prior to the early 2000s, genetic selection in the dairy industry primarily focused on milk yield or milk components [7–8]. Selection for female fertility began with the inclusion of daughter pregnancy rate in 2003 [9], and expanded to include overall heifer conception rate and CCR as well as conception rates to first breeding in 2009 [10]. Additional fertility measures such as early first calving (the age at which a heifer calves) were added to selection indexes in 2019 [11]. 

Since its inclusion in the Council for Dairy Cattle Breeding genomic evaluations, cow conception rate to the first breeding (CCR1) has improved by 7% points reaching 44%, while CCR to any service has improved by 6% points reaching 42% [4]. This improvement in CCR has been achieved through genomic selection using single nucleotide polymorphisms (SNPs) that are in linkage disequilibrium (LD) with the mutation responsible for the phenotype (causal mutation). To date, few casual variants are known or used in genomic selection for female fertility traits as finding the causal mutation can be both time consuming and challenging. The advantages to identifying the causal mutations is that the accuracy of prediction of fertility will be higher because the prediction is not reliant on the lack of changing LD with successive generations of meiotic events or recombination differences between breeds. Identifying the causal mutation also offers insights into the molecular mechanism of fertility [12]. 

Given the importance of fertility to the dairy industry and the lack of characterization of casual mutations associated with CCR, the goals of the current study were to: (1) identify leading edge genes and gene sets enriched for CCR and the number of times a cow was bred to achieve a pregnancy (TBRD) using gene set enrichment analysis using SNPs (GSEA-SNP) and (2) perform an association analysis of loci validated for an association with fertility (from at least two independent studies) using a custom genotype by sequencing (GBS) panel to identify putative causal variants associated with the CCR and TBRD in Holstein cows.

## Materials and methods

### Study population

This study was conducted with the approval of Washington State University’s Institutional Animal Care and Use Committee (#4295) and the authors confirm that this study is reported in accordance with ARRIVE guidelines. The Holstein study population consisted of 2,015 Holstein cows sampled from six dairies located in central Washington [13]. All dairies provided written agreement to participate in the study. No animals were euthanized for this study. Whole blood (~ 16 ml) was collected into EDTA tubes from each cow via venipuncture of the tail vein. Blood was collected from cows without being anesthetized, as approved by the Washington State University’s Institutional Animal Care and Use Committee (#4295). Cows (*n* = 951) were removed from the study if they experienced an inflammatory event or disease that could potentially have impacted pregnancy retention such as: lameness, fever, mastitis, uterine disease, spontaneous abortions, dystocia, foot disease, pink eye, respiratory disease, and metabolic issues. All cows were artificially inseminated (AI) at either observed estrus or using timed AI. Pregnancy status was determined at 35 days post insemination by rectal palpation of uterine contents. Pregnancy status after d35 was not evaluated for the current study, as this study aims to focus on early embryonic loss not fetal losses. Animals remaining in the study (*n* = 1064) were genotyped using the Illumina BovineHD Beadchip (San Diego, CA) at Neogen Laboratories (Lincoln, NE). Prior to any analysis, samples were quality control filtered for duplicates and individual call rate (< 90%) with 32 cattle removed. This left a total of 1,032 cows for the study: 494 cows that conceived to the first service, 472 cows that were bred 2–20 times before conceiving, and 66 infertile cows that never conceived after a minimum of 6 AI services (range 6–20 AI attempts) [13]. The phenotypes of CCR1 and TBRD were used for the GSEA-SNP and the association analysis. Phenotypes were coded as follows: CCR1 using 1 (successful conception to first service) and 0 (no conception at first service); and TBRD which used a scale from 1 to 20 depending on the number of inseminations a cow received.

For this study, only first lactation (first parity) cow records were used to ensure the entire study population were the same age for comparison. This data was collected retrospectively through farm management records. The average 305-day milk yields did not differ (*P* = 0.14) across the different phenotypic groups of cattle. The average 305-day milk yield for the entire population was 28,831 kg, additional milk yield information can be found in Supplemental Table 1. Conception rate did not differ between AI technicians (*P* > 0.05) or between service sires (*P* = 0.99) [13]. A total of 435 sires (433 Holstein and 2 Angus bulls) were used across all dairies with an average conception rate of 26.8% [13]. Most cows that failed to conceive to one sire were subsequently rebred to a different service sire reducing the risk for a cow to be categorized as infertile in the TBRD phenotype due solely to a service sire effect. Dairy of origin did have an effect on CCR1 and TBRD (*P* < 1 × 10^− 10^) and was included as a covariate in all analyses.

### Gene-set enrichment analysis-SNP

The genotypes from the Illumina BovineHD Beadchip (San Diego, CA) at Neogen Laboratories (Lincoln, NE), were used to conduct an association analysis to identify the SNPs that would serve as representatives for the genes in the gene sets [13]. A total of 625,093 SNP were analyzed and mapped to 21,039 protein-coding genes within the ARS-UCD 1.2 genome assembly (https://www.animalgenome.org/repository/cattle/UMC_bovine_coordinates/). SNPs with the greatest evidence of an association with CCR1 or TBRD were used as the gene proxies for the GSEA-SNP. One SNP represented each gene within a 17 kb region up- and down-stream of the gene. This 17 kb region was based on the average haplotype block size of a large (*n* = 4,800) Holstein cattle population [13–15] also genotyped with the BovineHD BeadChip calculated using the method described by Gabriel et al., in 2002 [16]. A single SNP could represent more than one gene if it fell within the parameters outlines above. Genes were ranked by their significance (*P*-value) for their association with CCR1 or TBRD from the genome wide association analysis.

The GSEA-SNP was performed following the methods of Wang et al. (2007) [17] and five gene set databases were used: Biocarta (217 gene sets; http://www.genecarta.com/), Gene Ontology or GO (3,147 gene sets; http://www.geneontology.com), Kyoto Encyclopedia of Genes and Genomes or KEGG (186 gene sets; http://www.genome.jp.kegg), Protein Analysis Through Evolutionary Relationships or PANTHER (165 gene sets; http://www.pantherdb.org), and Reactome (674 gene sets; http://www.reactome.org). An enrichment score for each gene set was computed using the highest value from the running sum statistics, similar to a weighted Kolmogorov-Smirnov-like statistic [18]. Each gene set received a permuted *P*-value calculated using 10,000 phenotype-based permutations in GenABEL in R [19]. To account for the varying number of genes within each gene set, a normalized enrichment score (NES) was computed, and enriched gene sets were those that had an NES ≥ 3.0. For enriched gene sets, a list of genes that contributed to the peak NES were identified as leading edge genes (LEG).

### Genotype by sequencing

Fertility validated loci were identified by comparing results from twenty-six studies [13, 15, 20–43] (Table 1), as well as the GSEA-SNP from the current study, that utilized a range of fertility phenotypes and cattle breeds. A locus was defined as SNP associated with fertility that were in linkage disequilibrium (LD) with each other (D’ > 0.75). This D’ threshold was used as it falls within ranges previously used to define SNPs within a locus [42]. To identify putative causal mutations in the 202 loci validated by two or more fertility studies, a custom genotyping array was designed after whole genome sequencing was conducted on 24 Holsteins at 10X coverage using the Illumina HiSeq X Ten at the University of Missouri sequencing core. Sequences of the 202 loci were mapped to the ARS-UCD 1.2 reference assembly. There were 5,102 variants identified within the 202 loci. Variants were screened to determine which ones were located in regulatory regions (*n* = 317), exons (*n* = 633), splice sites (*n* = 22), and evolutionarily conserved regions (*n* = 392). After screening, 1,358 variants within 152 loci were sent for probe design at Neogen Laboratories (Lincoln, NE). Of the variants identified, 284 variants (21%) failed the probe design due to the presence of secondary structures, high or low GC content, or repetitive motifs of the targeted sequence. Probes were successfully designed for 713 variants covering both directions and 361 variants where probes were successfully designed for a single direction. This resulted in the creation of a genotyping array with 1,787 probes which covered 100 of the 152 loci identified across studies. Paired-end 150 bp libraries for 1,064 cows were prepared and analyzed at Neogen laboratories (Lincoln, NE) following Tecan’s targeted genotyping V2 Allegro protocols (Männedorf, Switzerland). Sequencing was performed using an Illumina NextSeq2000 system (San Diego, CA).

**Table 1:** List of cattle fertility studies used for loci validation

Study^1^	Phenotype(s)^2^	Breeds^3^
Akanno et al. 2015 [31]	AFC, PR	Crossbred beef^Ca^
Blaschek et al. 2011 [24]	Non-compensatory fertility	Holstein^Ur^
Cochran et al. 2013 [27]	DPR	Holstein^Ur^
Cole et al. 2011 [25]	DPR	Holstein^U^
Fonseca et al. 2018 [41]	Reproduction	Brangus^U^, Brahman^A^, Tropical Composite^A^
Galliou et al. 2020 [42]	HCR1, TBRD	Holstein^U^
Höglund et al. 2009 [20]	AISC, AISH, ICF, IFLC, IFLH, NRRC, NRRH	Holstein^N^
Höglund et al. 2014 [30]	AISC, AISH, FTI, ICF, IFLC, IFLH, NRRC, NRRH	Holstein^N^, Jersey^N^, Nordic Red^N^
Höglund et al. 2015 [32]	AISC, AISH, FTI, ICF, IFLC, IFLH, NRRC, NRRH	Nordic Red^N^
Huang et al. 2010 [21]	FR, BR	Holstein^U^
Iso-Touru et al. 2016 [33]	Milk production, AISC, AISH, FTI, ICF, IFLC, IFLH, NRRC, NRRH	Nordic Red^N^
Kiser et al. 2019a [13]	CCR1, TBRD	Holstein^U^
Kiser et al. 2019b [15]	HCR1, TBRD	Holstein^U^
Liu et al. 2017	AFS, AISC, CCR, DO, ICF, IFLC, NRRC	Holstein^C, N^
Minozzi et al. 2013 [28]	CI, DFS, FTI, NRR	Holstein^It^
Minten et al. 2013 [29]	High vs. Low Fertile	Crossbred Beef^U^
Moore et al. 2016 [34]	CI	Holstein^A, I^, Jersey^A^
Müller et al. 2017 [38]	CTFS, DO, FSTCC, FSTCH, NRRC, NRRH,	Holstein^G^
Nayeri et al. 2016 [35]	CTFS, DO, FSTCH	Holstein^Ca^
Neupane et al. 2017 [39]	P28	Angus crosses^U^
Oliver et al. 2020 [43]	HCR1, TBRD	Crossbred Beef^U^
Olsen et al. 2011 [26]	NRRC, NRRH	Nordic Red^N^
Ortega et al. 2016 [36]	DPR	Holstein^U^
Ortega et al. 2017 [40]	DPR	Holstein^U^
Pryce et al. 2010 [22]	Fertility	Holstein^A^, Jersey^A^
Sahana et al. 2010 [23]	AISC, AISH, FTI, IFLC, IFLH, ICF, NRRC, NRRH	Holstein^N^

^1^The citation number for each study is listed in superscript brackets. ^2^Traits abbreviated as follows: AFC - age at first calving; AISC - number of inseminations to conception in cows; AISH - number of inseminations to conception in heifers; AFS - age at first insemination; BR - blastocyst rate; CCR1 - conception rate to first insemination in cows; CI - calving interval; CTFS - days from calving to first insemination; DFS - days to first service; DO - days open; DPR - daughter pregnancy rate; FSTCC - days from first service to conception in cows; FSTCH - days from first service to conception in heifers; FR - fertility rate; FTI - fertility index; HCR - heifer conception rate; HCR1 - conception rate to first insemination in heifers; ICF - interval (in days) from calving to first insemination; IFLC - days from first to last insemination in cows; IFLH - days from first to last insemination in heifers; NRR − 56 day non return rate; NRRC − 56 day non return rate in cows; NRRH − 56 day non return rate in heifers; P28- pregnancy success at day 28 post embryo transfer; P42 - pregnancy success within first 42 days of mating; PR - pregnancy rate; TBRD - number of times bred to conception. ^3^Cattle breeds loci were previously identified are listed with the country or region the population was from indicated in superscript as follows: Australia - A; Canada - Ca; Chinese - C; Germany - G; Ireland - I; Nordic - N; United States - U; Unreported - Ur

**Fig. 1:**
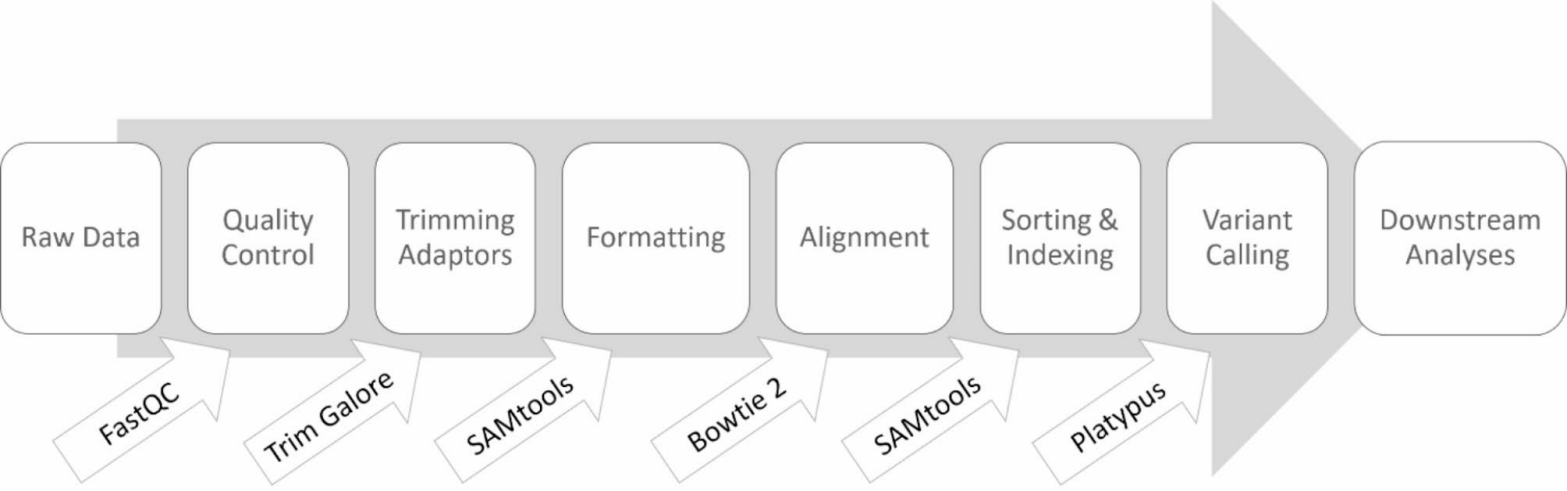
Processing pipeline for GBS data

Raw sequencing data was processed to create variant call format (VCF) files for the association analysis (Fig. 1). Raw sequencing reads were assessed for quality using the FastQC software [44] and adaptors were trimmed off with Trim Galore and Cutadapt [45, 46]. Files were aligned to the ARS-UCD 1.2 reference assembly using bowtie2 [47], and sorted and indexed using SAMtools [48]. Variant calling was successful for 1,222 or the 1,787 probes using Platypus [49]. The Platypus VCF files were then uploaded into SNP and Variation Suite (SVS) version 8.9.1 (Golden Helix, Bozeman, MT) for analysis. Quality control filtering of SNPs was performed on the 1,222 called variants. SNPs were then removed for call rate < 85% (*n* = 127) or minor allele frequency < 1% (*n* = 80), leaving 1,015 SNPs spanning 100 loci that were further analyzed.

### Association analysis

Three association analyses were conducted for each phenotype (CCR1 and TBRD) using the GBS variants in conjunction with the Illumina BovineHD BeadChip (San Diego, CA) genotypes the cattle were originally genotyped on. Given that the model of inheritance for cow fertility is unknown, three modes of inheritance were analyzed for each phenotype: additive, dominant, and recessive. SNPs were considered significant if their uncorrected *P*-value met the Wellcome Trust significance threshold of *P* < 1 × 10^− 5^ [50]. The association analyses were performed using SVS software and an efficient mixed-model association expedited (EMMAX) model [51]. This model was described in detail previously [13, 15, 51]. Briefly, the model is described by$\:\:\varvec{y}=\mathbf{X}\varvec{\upbeta\:}+\varvec{Z}u+\text{ϵ}$, **y** is the *n* × 1 vector of observed phenotypes, **X** is an *n* × *f* matrix of fixed effects (*f*), **β** is an *f* × 1 vector containing the fixed effect coefficients, **Z** is an *n* × *t* matrix relating the random effects (*t*) to the phenotype, and $\:u$ is the random effect of the mixed model. The model assumes residuals to be independent with an identical distribution such that $\:Var\left(u\right)=\:{{\sigma\:}_{g}}^{2}\varvec{K}$ and $\:\left(ϵ\right)=\:{{\sigma\:}_{e}}^{2}\varvec{I}$, and$\:\:Var\left(y\right)=\:{{\sigma\:}_{g}}^{2}\varvec{Z}\varvec{K}{\varvec{Z}}^{\varvec{{\prime\:}}}+\:{{\sigma\:}_{e}}^{2}\varvec{I}$. For this study, **K** is a matrix of pairwise genomic relationships and **Z** is the identity matrix, **I** [52]. 

Observed heritability was estimated using a genomic best linear unbiased predictor (GBLUP) [53] analysis using AI-REML (average information algorithm) [54, 55]. While computationally intensive, this method to estimate heritability provides a more accurate estimation of heritability when sample size is limited.

## Results

### Gene-set enrichment analysis-SNP

The GSEA-SNP identified 95 gene sets with NES ≥ 3 (Supp. Table 2) that were enriched for CCR1. There were 1,473 unique LEG across the 95 gene sets, with 2 LEG, scinderin (*SCIN*) and villin 1 (*VIL1*), found in 40 of the 95 gene sets. Gene sets with greatest evidence for enrichment with CCR1 (NES > 4.0) are shown in Table 2. For TBRD, 67 gene sets were enriched (NES ≥ 3; Supp. Table 3) and 4 gene sets had an NES ≥ 4 (Table 3). Two gene sets with NES > 4.0 for TBRD (recruitment of mitotic centrosome proteins and complexes; loss of NLP from mitotic centrosomes) also were enriched for CCR1 with NES > 4.0. There were 1,438 unique LEG for TBRD. A single LEG, Rac family small GTPase 1 (*RAC1*), was shared in 28 gene sets.

**Table 2:** Gene set enrichment analysis– single nucleotide polymorphism results for conception rate to first service (CCR1) in Holstein cows

Gene Sets^a^	# Genes (# LEG)^b^	NES^c^	Leading Edge Genes^d^
Cell Cycle, Mitotic (R-HSA-69278)	300 (93)	5.08	*RAD21*,* PAFAH1B1*,* CEP290*,* CLIP1*,* RBL2*,* TUBB4B*,* CENPH*,* CCNB1*,* CDK6*,* CCNE2*,* CEP250*,* CENPJ*,* CASC5*,* PRIM2*,* KNTC1*,* CDK1*,* YWHAE*,* CLASP1*,* BTRC*,* CDC14A*,* SDCCAG8*,* CDK7*,* CDKN2A*,* CDKN2B*,* CEP41*,* CCNA2*,* NUF2*,* KIF23*,* PSMA4*,* PSMB8*,* PSMB9*,* MCM7*,* MCM3*,* MCM2*,* UBE2C*,* OFD1*,* RPA3*,* KIF2B*,* NUP133*,* AHCTF1*,* PRKAR2B*,* NUDC*,* PRIM1*,* TYMS*,* ALMS1*,* CENPT*,* ANAPC5*,* CEP72*,* CEP70*,* CUL1*,* DHFR*,* ANAPC7*,* SKA1*,* AKAP9*,* NINL*,* PRKACA*,* STAG2*,* CDC45*,* CCNE1*,* PLK1*,* PSMD11*,* DYRK1A*,* CDT1*,* PSMB7*,* ITGB3BP*,* MAD1L1*,* NUMA1*,* INCENP*,* CEP135*,* RBL1*,* FBXO5*,* PPP2R5E*,* ANAPC10*,* DCTN1*,* Sect. 13*,* PSMB10*,* CDC7*,* PTTG1*,* BUB1B*,* CENPP*,* BUB1*,* PSMA2*,* LIN52*,* PSME1*,* PSME2*,* TUBGCP3*,* FGFR1OP*,* PSMB2*,* PPP2R2A*,* PSMC1*,* RB1*,* YWHAG*,* CCND2*
Cell Cycle (R-HSA-1640170)	364 (126)	4.87	*RAD21*,* PAFAH1B1*,* CEP290*,* CLIP1*,* RBL2*,* TUBB4B*,* CENPH*,* CCNB1*,* CDK6*,* RFWD2*,* SYNE2*,* H2AFX*,* CCNE2*,* CEP250*,* CENPJ*,* CASC5*,* PRIM2*,* KNTC1*,* CDK1*,* YWHAE*,* CLASP1*,* BTRC*,* CDC14A*,* SDCCAG8*,* CDK7*,* CDKN2A*,* CDKN2B*,* CEP41*,* CCNA2*,* NUF2*,* RUVBL1*,* KIF23*,* PSMA4*,* POT1*,* PSMB8*,* PSMB9*,* MCM7*,* MCM3*,* ATR*,* MCM2*,* UBE2C*,* OFD1*,* HIST1H4D*,* RPA3*,* KIF2B*,* NUP133*,* AHCTF1*,* PRKAR2B*,* SYNE1*,* NUDC*,* PRIM1*,* TYMS*,* ALMS1*,* CENPT*,* ANAPC5*,* CEP72*,* CEP70*,* CUL1*,* FKBP6*,* DHFR*,* ANAPC7*,* SKA1*,* HIST1H2BN*,* SMC1B*,* AKAP9*,* NINL*,* PRKACA*,* STAG2*,* CDC45*,* CCNE1*,* PLK1*,* PSMD11*,* DYRK1A*,* MIS18A*,* RAD1*,* CDT1*,* PSMB7*,* ITGB3BP*,* MAD1L1*,* NUMA1*,* INCENP*,* HUS1*,* CEP135*,* RBL1*,* FBXO5*,* PPP2R5E*,* ANAPC10*,* DCTN1*,* Sect. 13*,* PSMB10*,* CDC7*,* PTTG1*,* BUB1B*,* CENPP*,* BUB1*,* PSMA2*,* LIN52*,* PSME1*,* PSME2*,* TUBGCP3*,* FGFR1OP*,* PSMB2*,* PPP2R2A*,* PSMC1*,* RB1*,* YWHAG*,* CCND2*,* HIST1H2BI*,* REC8*,* CEP76*,* HJURP*,* CHEK2*,* HIST1H2BJ*,* PSMC6*,* HIST3H2BB*,* RSF1*,* GINS4*,* MCM8*,* ORC2*,* PCNT*,* LIN54*,* ANAPC1*,* PSMC2*,* HIST1H2BB*,* ANAPC2*,* SSNA1*
Protein Polymerization (GO:0051258)	79 (21)	4.81	*TWF1*,* TUBB4B*,* ARFIP2*,* TUBA8*,* CAPZA2*,* F2*,* SCIN*,* WASF1*,* SLAIN2*,* ANG*,* VIL1*,* CAPZB*,* TMSB4*,* RASA1*,* SNCA*,* ARPIN*,* ARPC1A*,* UBE2C*,* RAC1*,* WASL*,* ARPC5*
Recruitment of Mitotic Centrosome Proteins and Complexes (R-HSA-380270)	62 (22)	4.77	*PAFAH1B1*,* CEP290*,* TUBB4B*,* CCNB1*,* CEP250*,* CENPJ*,* CDK1*,* YWHAE*,* CLASP1*,* SDCCAG8*,* CEP41*,* OFD1*,* PRKAR2B*,* ALMS1*,* CEP72*,* CEP70*,* AKAP9*,* NINL*,* PRKACA*,* PLK1*,* NUMA1*,* CEP135*
Mitotic G2-G2/M phases (R-HSA-453274)	76 (24)	4.74	*PAFAH1B1*,* CEP290*,* TUBB4B*,* CCNB1*,* CEP250*,* CENPJ*,* CDK1*,* YWHAE*,* CLASP1*,* SDCCAG8*,* CDK7*,* CEP41*,* CCNA2*,* OFD1*,* PRKAR2B*,* ALMS1*,* CEP72*,* CEP70*,* AKAP9*,* NINL*,* PRKACA*,* PLK1*,* NUMA1*,* CEP135*
Loss of NLP From Mitotic Centrosomes (R-HSA-380259)	55 (20)	4.68	*PAFAH1B1*,* CEP290*,* TUBB4B*,* CEP250*,* CENPJ*,* CDK1*,* YWHAE*,* CLASP1*,* SDCCAG8*,* CEP41*,* OFD1*,* PRKAR2B*,* ALMS1*,* CEP72*,* CEP70*,* AKAP9*,* NINL*,* PRKACA*,* PLK1*,* CEP135*
Cellular Component Disassembly (GO:0022411)	81 (20)	4.64	*TWF1*,* DPP4*,* MICAL3*,* CCNB1*,* GABARAPL1*,* MMP13*,* CAPZA2*,* SCIN*,* VIL1*,* CAPZB*,* ENDOG*,* FOXL2*,* BAX*,* KIF2B*,* FIS1*,* NAPB*,* STMN1*,* DSTN*,* SBDS*,* MTRF1L*
Macromolecular Complex Assembly (GO:0065003)	372 (84)	4.47	*TWF1*,* SRSF1*,* TUBB4B*,* CNOT7*,* HES1*,* SF3A1*,* H1FOO*,* CENPH*,* CHMP4A*,* DPAGT1*,* CAMK2D*,* KCTD1*,* SAMHD1*,* VAMP4*,* ARFIP2*,* RAD51*,* SRR*,* TUBA8*,* SLC6A4*,* CAPZA2*,* TBCA*,* OAT*,* F2*,* FECH*,* SCIN*,* WASF1*,* SLAIN2*,* ANG*,* VIL1*,* PEX5*,* VWF*,* STRAP*,* EIF6*,* COX7A2L*,* CAPZB*,* SNAP29*,* MGST1*,* TMSB4*,* RASA1*,* IGF1R*,* GEMIN8*,* LRRC6*,* NAP1L1*,* CBR4*,* SNCA*,* NUDT21*,* ARPIN*,* COX19*,* ARPC1A*,* ASF1A*,* UBE2C*,* NLRC4*,* NDUFAF6*,* MICU1*,* CLU*,* BAX*,* LMO4*,* RAC1*,* CAT*,* WASL*,* ARPC5*,* FIS1*,* NAPB*,* PRPF19*,* CYBA*,* SBDS*,* DRC1*,* LONP1*,* ARPC3*,* TARBP2*,* PDCL*,* IL5*,* H4*,* HIST1H1A*,* ATP6V0A2*,* PSMD11*,* RDX*,* ARL6*,* MIS18A*,* FAS*,* LUC7L3*,* KCTD5*,* SAR1A*,* MIF*
Signaling by NGF (R-HAS-9031628)	207(80)	4.42	*NTRK2*,* SOS2*,* APH1B*,* RAP1A*,* PRKCE*,* ADCY2*,* ADCY5*,* ATF1*,* VAV3*,* MAPK8*,* AKT3*,* MYD88*,* SORCS3*,* CDK1*,* AKAP13*,* ADAM17*,* YWHAE*,* MCF2*,* TIAM2*,* CAMK4*,* TIAM1*,* PRKCI*,* FOXO3*,* ADCY1*,* DUSP6*,* FGD2*,* ADCY9*,* SQSTM1*,* FGD4*,* ABR*,* PIK3R1*,* NR4A1*,* ITSN1*,* PDPK1*,* OBSCN*,* RALB*,* MEF2A*,* PDE1A*,* AP2A1*,* PRKAR2A*,* RAC1*,* RPS6KA3*,* CHUK*,* KIDINS220*,* AP2B1*,* PRKAR2B*,* FURIN*,* ADCYAP1R1*,* PIK3CB*,* RASGRF2*,* ARHGEF3*,* SRC*,* ARHGAP4*,* MAPKAP1*,* PHLPP1*,* SHC3*,* CASP9*,* FGD3*,* LINGO1*,* ARHGEF6*,* PRKACA*,* MAP2K5*,* ITPR3*,* ADCY8*,* ADCY7*,* ITGB3BP*,* MEF2C*,* TRIO*,* FOXO1*,* PLCG1*,* AKT1S1*,* MTOR*,* PIK3CA*,* CLTC*,* CLTA*,* RIPK2*,* ADCY3*,* TRAF6*,* PRKAR1B*,* APH1A*
Protein Complex Assembly (GO:0065003)	303 (73)	4.38	*TWF1*,* TUBB4B*,* HES1*,* H1FOO*,* CENPH*,* CHMP4A*,* DPAGT1*,* CAMK2D*,* KCTD1*,* SAMHD1*,* VAMP4*,* ARFIP2*,* RAD51*,* SRR*,* TUBA8*,* SLC6A4*,* CAPZA2*,* TBCA*,* OAT*,* F2*,* SCIN*,* WASF1*,* SLAIN2*,* ANG*,* VIL1*,* PEX5*,* VWF*,* COX7A2L*,* CAPZB*,* SNAP29*,* MGST1*,* TMSB4*,* RASA1*,* IGF1R*,* LRRC6*,* NAP1L1*,* CBR4*,* SNCA*,* NUDT21*,* ARPIN*,* COX19*,* ARPC1A*,* ASF1A*,* UBE2C*,* NLRC4*,* NDUFAF6*,* MICU1*,* CLU*,* BAX*,* LMO4*,* RAC1*,* CAT*,* WASL*,* ARPC5*,* FIS1*,* NAPB*,* CYBA*,* DRC1*,* LONP1*,* ARPC3*,* PDCL*,* IL5*,* H4*,* HIST1H1A*,* ATP6V0A2*,* PSMD11*,* RDX*,* ARL6*,* MIS18A*,* FAS*,* KCTD5*,* SAR1A*,* MIF*
Regulation of RNA Splicing (GO:0043484)	33 (13)	4.31	*SRSF1*,* SF3A1*,* RBFOX1*,* CLK3*,* SNW1*,* HNRNPF*,* SNRNP70*,* PIK3R1*,* RBM22*,* CELF3*,* RBFOX2*,* PRPF19*,* NSRP1*
Actin Filament Polymerization (GO:0030041)	48 (15)	4.29	*TWF1*,* ARFIP2*,* CAPZA2*,* SCIN*,* WASF1*,* ANG*,* VIL1*,* CAPZB*,* TMSB4*,* RASA1*,* ARPIN*,* ARPC1A*,* RAC1*,* WASL*,* ARPC5*
Actin Polymerization or Depolymerization (GO:0008154)	58 (17)	4.27	*TWF1*,* MICAL3*,* ARFIP2*,* CAPZA2*,* SCIN*,* WASF1*,* ANG*,* VIL1*,* CAPZB*,* TMSB4*,* RASA1*,* ARPIN*,* ARPC1A*,* RAC1*,* WASL*,* ARPC5*,* DSTN*
Cilium Morphogenesis (GO:0060271)	46 (8)	4.24	*CEP290*,* ASAP1*,* IFT46*,* IFT43*,* TTLL1*,* C7H5ORF30*,* CEP41*,* LRRC6*
Protein Complex Biogenesis (GO:0070271)	306 (73)	4.23	*TWF1*,* TUBB4B*,* HES1*,* H1FOO*,* CENPH*,* CHMP4A*,* DPAGT1*,* CAMK2D*,* KCTD1*,* SAMHD1*,* VAMP4*,* ARFIP2*,* RAD51*,* SRR*,* TUBA8*,* SLC6A4*,* CAPZA2*,* TBCA*,* OAT*,* F2*,* SCIN*,* WASF1*,* SLAIN2*,* ANG*,* VIL1*,* PEX5*,* VWF*,* COX7A2L*,* CAPZB*,* SNAP29*,* MGST1*,* TMSB4*,* RASA1*,* IGF1R*,* LRRC6*,* NAP1L1*,* CBR4*,* SNCA*,* NUDT21*,* ARPIN*,* COX19*,* ARPC1A*,* ASF1A*,* UBE2C*,* NLRC4*,* NDUFAF6*,* MICU1*,* CLU*,* BAX*,* LMO4*,* RAC1*,* CAT*,* WASL*,* ARPC5*,* FIS1*,* NAPB*,* CYBA*,* DRC1*,* LONP1*,* ARPC3*,* PDCL*,* IL5*,* H4*,* HIST1H1A*,* ATP6V0A2*,* PSMD11*,* RDX*,* ARL6*,* MIS18A*,* FAS*,* KCTD5*,* SAR1A*,* MIF*
Macromolecular Complex Binding (GO:0044877)	297 (97)	4.13	*PAFAH1B1*,* RAP1A*,* H1FOO*,* PPARGC1A*,* CENPH*,* GABARAPL1*,* MMP13*,* MUM1*,* SLC6A4*,* YWHAE*,* PLS1*,* MYO10*,* F2*,* STRN3*,* COLEC12*,* SCIN*,* WASF1*,* SEC61A2*,* MYB*,* HMGB4*,* TDRD3*,* GNAO1*,* VIL1*,* VWF*,* EIF6*,* PRKCB*,* CAPZB*,* GNAI1*,* TFAM*,* POLR3A*,* IGF1R*,* SEC61A1*,* DNAJC2*,* HMGN4*,* PIK3R1*,* USH1C*,* PDGFA*,* MEF2A*,* ARPC1A*,* ASF1A*,* PRIMPOL*,* LMO2*,* MYO1D*,* NANOG*,* MTA3*,* CCNT1*,* TNNC1*,* MTM1*,* KIF2B*,* MSR1*,* MEN1*,* KDM8*,* ZNHIT1*,* PPIH*,* SCARB1*,* SBDS*,* NR5A1*,* CDC5L*,* SPARC*,* PLAC8*,* ARPC3*,* MSH2*,* SKA1*,* RNF169*,* FBLN5*,* RNF20*,* HIST1H1A*,* THBS4*,* GNAT3*,* ITGB6*,* PLK1*,* BAP1*,* RBPJ*,* ANKRD54*,* CETN1*,* FOXP1*,* KCTD5*,* MEF2C*,* FSCN2*,* UHRF1*,* MCMBP*,* HMGN3*,* GNB1*,* ERMN*,* GNAT1*,* REEP4*,* MAP1LC3B*,* ASPN*,* CITED2*,* AAK1*,* TMOD1*,* ITGB1*,* ARPC1B*,* URI1*,* PPARG*,* SPDL1*,* MYL12A*
Cellular Protein Complex Disassembly (GO:0032984)	40 (13)	4.06	*TWF1*,* MICAL3*,* CCNB1*,* CAPZA2*,* SCIN*,* VIL1*,* CAPZB*,* KIF2B*,* NAPB*,* STMN1*,* DSTN*,* SBDS*,* MTRF1L*
Cellular Macromolecular Complex Assembly (GO:0006461)	245 (55)	4.06	*TWF1*,* SRSF1*,* TUBB4B*,* CNOT7*,* SF3A1*,* H1FOO*,* CENPH*,* VAMP4*,* ARFIP2*,* TUBA8*,* CAPZA2*,* TBCA*,* F2*,* SCIN*,* WASF1*,* SLAIN2*,* ANG*,* VIL1*,* STRAP*,* EIF6*,* COX7A2L*,* CAPZB*,* SNAP29*,* TMSB4*,* RASA1*,* GEMIN8*,* LRRC6*,* NAP1L1*,* SNCA*,* ARPIN*,* COX19*,* ARPC1A*,* ASF1A*,* UBE2C*,* NDUFAF6*,* RAC1*,* WASL*,* ARPC5*,* NAPB*,* PRPF19*,* CYBA*,* SBDS*,* DRC1*,* LONP1*,* ARPC3*,* TARBP2*,* H4*,* HIST1H1A*,* ATP6V0A2*,* PSMD11*,* RDX*,* ARL6*,* MIS18A*,* LUC7L3*,* SAR1A*
Regulation of Actin Filament Polymerization (GO:0030833)	44 (6)	4.03	*TWF1*,* CAPZA2*,* SCIN*,* VIL1*,* CAPZB*,* TMSB4*

^a^ Accession code for each gene set is in parentheses: Reactome– R; Gene Ontology– GO. ^b^ Total number of genes included in a gene set, with the number of leading edge genes (LEG) listed in parentheses. ^c^ Normalized enrichment score (NES) for each gene set, calculated by Kolmogorov-Smirnov-like statistics. ^d^ Leading edge gene identified by each gene set, listed in order of significance and identified by gene symbols as listed in the National Center for Biotechnology Information gene database (https://www.ncbi.nlm.nih.gov/gene/; accessed: 9 January, 2022)

**Table 3:** Gene set enrichment analysis– single nucleotide polymorphism results for number of breeding services required to conceive (TBRD) in Holstein cows

Gene Sets^a^	# Genes (# LEG)^b^	NES^c^	Leading Edge Genes^d^
Recruitment of mitotic centrosome proteins and complexes (R-HSA-380270)	62 (13)	4.327	*TUBB4B*,* CEP290*,* PAFAH1B1*,* YWHAE*,* CEP250*,* CCNB1*,* CDK1*,* CENPJ*,* PLK1*,* CSNK1D*,* SDCCAG8*,* CLASP1*,* PRKAR2B*
Loss of NLP from mitotic centrosomes (R-HSA-380259)	55 (12)	4.293	*TUBB4B*,* CEP290*,* PAFAH1B1*,* YWHAE*,* CEP250*,* CDK1*,* CENPJ*,* PLK1*,* CSNK1D*,* SDCCAG8*,* CLASP1*,* PRKAR2B*
Cellular component assembly (GO:0022607)	515 (173)	4.08	*NDOR1*,* TUBB4B*,* TWF1*,* SF3A1*,* SRSF1*,* RAP1A*,* HES1*,* SAMHD1*,* IFT46*,* H1FOO*,* CAMK2D*,* KCTD1*,* SNAP29*,* CNOT7*,* CENPH*,* SERPINF2*,* SLC6A4*,* CHMP4A*,* EIF6*,* DPAGT1*,* CAPZA2*,* TMSB4*,* COX7A2L*,* WASF1*,* TUBA8*,* NRXN3*,* UBE2C*,* ASF1A*,* GABARAPL1*,* ANG*,* PRPF19*,* MYO10*,* NUDT21*,* TMEM231*,* FECH*,* MEF2A*,* SLAIN2*,* VAMP4*,* RASA1*,* ATPAF2*,* CSRP3*,* NDUFAF6*,* GTPBP8*,* MGST1*,* RAC2*,* TMEM216*,* CSNK1D*,* RAD51*,* SRR*,* CYBA*,* MAP1LC3A*,* PEX5*,* CRTC2*,* ATP6V0D1*,* ARPC3*,* OGFOD1*,* ITGB1BP1*,* ARPC5L*,* TTLL1*,* LSM14A*,* KCTD19*,* FBLN5*,* COX19*,* VIL1*,* TBCA*,* IL5*,* LUC7L3*,* SNAP25*,* KCTD5*,* ATL1*,* PDCL*,* STRAP*,* PSMD11*,* BIRC5*,* PPP1R16B*,* VWF*,* ARFIP2*,* SRPX2*,* GAP43*,* LMO4*,* NAPB*,* KCNB2*,* RAP1B*,* CAV1*,* CDC42EP2*,* SHMT1*,* SLU7*,* MPP7*,* CAPZB*,* ATP6V0A2*,* GPM6A*,* CEP41*,* MEF2C*,* F2*,* MIS18A*,* IGF1R*,* UPK1A*,* MAPT*,* LCMT1*,* PRKACA*,* PSMG2*,* PSMD9*,* RAPGEF2*,* ARPIN*,* IFT20*,* CAT*,* EIF3G*,* CAPN3*,* EMP2*,* CLSTN3*,* FOXP1*,* NDUFS4*,* DRC1*,* KCTD18*,* SPICE1*,* ACTG1*,* ATG3*,* UBE2S*,* PICK1*,* APP*,* TBCD*,* GRB7*,* WASL*,* CCDC151*,* TBC1D7*,* ATP6V1D*,* FIS1*,* ARPC1A*,* SNRPE*,* KIT*,* CAND1*,* S100A10*,* PAK1*,* TMEM138*,* DDX39B*,* CLGN*,* TCAP*,* RAC1*,* APOA1*,* NAP1L1*,* RDX*,* HAUS1*,* NAP1L4*,* COX15*,* SAR1A*,* COG4*,* SCO2*,* MCIDAS*,* F2RL1*,* MMS19*,* DBNL*,* USO1*,* ATG5*,* TUBB2B*,* CHAF1A*,* TARBP2*,* SFRP1*,* DCXR*,* COL17A1*,* LONP1*,* NASP*,* KCNJ2*,* SNCA*,* SLC39A12*,* BRIX1*,* CFL2*,* NDUFAF4*,* PRKAR1A*,* MFAP4*,* SNRPF*,* VDAC2*,* SLC2A1*,* GEMIN8*
Regulation of RNA splicing (GO:0043484)	33 (9)	4.062	*SF3A1*,* SRSF1*,* RBFOX1*,* HNRNPF*,* PRPF19*,* PIK3R1*,* CLK3*,* NSRP1*,* SNW1*

^a^ Accession code for each gene set is in parentheses: Reactome– R; Gene Ontology– GO. ^b^ Total number of genes included in a gene set, with the number of leading edge genes (LEG) listed in parentheses. ^c^ Normalized enrichment score (NES) for each gene set, calculated by Kolmogorov-Smirnov-like statistics. ^d^ Leading edge gene identified by each gene set, listed in order of significance and identified by gene symbols as listed in the National Center for Biotechnology Information gene database (https://www.ncbi.nlm.nih.gov/gene/; accessed: 9 January, 2022)

Thirty-four gene sets were enriched in both CCR1 and TBRD (Table 4), which included 788 shared LEG. The three most commonly shared LEG were twinfilin actin binding protein 1 (*TWF1*) identified in 58 gene sets, *RAC1* in 59 gene sets and *VIL1* in 61 gene sets that contribute to the function of the cytoskeleton.

**Table 4:** List of gene sets enriched for both cow conception rate at first service (CCR1) and the number of breeding services required for conception (TBRD)

Functional Category	Gene set^a^	NES ^b^	
		CCR1	TBRD
*Cellular Component Organization*	Actin cytoskeleton organization (GO:0030036)	3.055	3.32
	Actin filament based process (GO:0030029)	3.101	3.245
	Cellular component assembly (GO:0022607)	3.34	4.08
	Cellular component biogenesis (GO:0044085)	3.261	3.626
	Cellular localization (GO:0051641)	3.765	3.083
	Cellular macromolecular complex assembly (GO:0065003)	4.055	3.434
	Cellular macromolecule localization (GO:0070727)	3.031	3.146
	Cellular protein complex assembly (GO:0065003)	3.932	3.35
	Cytoskeleton organization (GO:0007010)	3.331	3.183
	Gap junction (K - hsa04540)	3.966	3.5
	Macromolecular complex assembly (GO:0065003)	4.472	3.967
	Macromolecular complex binding (GO:0044877)	4.131	3.348
	Macromolecular complex subunit organization (GO:0043933)	3.261	3.785
	Macromolecule localization (GO:0033036)	3.051	3.057
	Organelle localization (GO:0051640)	3.509	3.15
	Protein complex assembly (GO:0065003)	4.384	3.633
	Protein complex biogenesis (GO:0070271)	4.234	3.488
*Mitotic Cell Cycle*	Cell cycle (R-HSA-1640170)	4.869	3.414
	Cell Cycle, Mitotic (R-HSA-69278)	5.077	3.831
	Cell division (GO:0051301)	3.092	3.649
	Loss of NLP from mitotic centrosomes (R-HSA-380259)	4.676	4.293
	Mitotic G2-G2/M phases (R-HSA-453274)	4.744	3.523
	Recruitment of mitotic centrosome proteins and complexes (R-HSA-380270)	4.769	4.327
*RNA & mRNA Metabolic Processes*	Regulation of mRNA metabolic process (GO:1903311)	3.423	3.318
	Regulation of mRNA processing (GO:0050684)	3.411	3.222
	Regulation of RNA splicing (GO:0043484)	4.311	4.062
*Signaling Pathways*	Neurotrophin signaling pathway (K - hsa04722)	3.749	3.293
	Notch signaling pathway (P00045)	3.139	3.629
	Signaling by NGF (R-HSA-187037)	4.423	3.208
*Other*	Cilium morphogenesis (GO:0060271)	4.236	3.237
	Positive regulation of lipid biosynthetic process (GO:0046889)	3.281	3.112
	Protein polymerization (GO:0051258)	4.806	3.422
	Retinol metabolism (K - map00830)	3.503	3.537
	Transmembrane transport of small molecules (R-HSA-382551)	3.43	3.23

^a^ Accession code for each gene set is in parentheses: Reactome– R; Gene Ontology– GO; Protein Analysis Through Evolutionary Relationships– P; Kyoto Encyclopedia for Genes and Genomes - K. ^b^ Normalized enrichment score (NES) for each gene set for CCR1 and TBRD

Many of the shared gene sets can be grouped into categories based on common functions. These groups include gene sets related to cellular component organization and structure (*n* = 17), mitotic cell cycle/mitosis (*n* = 6), RNA and mRNA metabolic processes (*n* = 3), and signaling pathways (*n* = 3). The remaining five gene sets have functions that do not fall in any of the above-mentioned categories (Table 4).

### Association analyses

There were no SNP associated with CCR1 in any model (*P* < 1 × 10^− 5^). However, a single locus on BTA1 at 146 Mb in the additive model had a false discovery rate (FDR) of 0.04, though the *P*-value did not meet the Wellcome Trust significance threshold. The observed heritability for this model was 0.19 ± 0.05.

The TBRD association analysis identified 4 SNP (3 loci), two on BTA1 and one on BTA20 in the additive model (*P* < 1 × 10^− 5^; Table 5). The locus on BTA1 at 146 Mb that was associated with TBRD was the same locus in the additive model of CCR1 that trended toward significance (FDR = 0.04). This locus contained a synonymous variant within disco interacting protein 2 homolog A (*DIP2A*). The other locus on BTA1 was located at 83 Mb. The two SNP within this locus were a missense and a synonymous variant within the first exon of *ENSBTAG00000032217*. This missense mutation (rs110205198), which results in an amino acid change from cysteine to serine, was further examined to determine if there were any predicted detrimental effects caused by the amino acid substitution. This was done using the sequencing homology-based tool SIFT (sorts intolerant from tolerant) which predicts if substitutions of amino acids could result in a phenotypic effect of the protein through a change in its structure with values ranging from 0 (intolerable change) to 1 (tolerated change) [56]. The predicted damage score for the amino acid substitution caused by this mutation was only 0.9 (with low confidence) indicating the substitution is likely tolerated. However, the low confidence warning indicates that there was not enough diversity between the sequences to accurately predict altered protein function [56]. The locus on BTA20 contained a variant within a CNV (nsv810323). No SNP were associated with TRBD in the dominant or recessive models and observed heritability for TBRD was 0.18 ± 0.05.

**Table 5:** Single nucleotide polymorphisms associated with cow fertility using GBS variants

BTA (Mb)^a^	# SNP in Locus	Phenotype^b^	Model^c^	GBS *P*-Value	Region Feature(s)^d^
1 (83.7)	2	CCR1 TBRD	Add Add	0.04* 2.10 × 10^− 11^	*ENSBTAG00000032217*
1 (146.1)	1	TBRD	Add	2.23 × 10^− 08^	*DIP2A*
20 (46.0)	1	TBRD	Add	1.82 × 10^− 06^	nsv810323

^a^ Chromosome location of the locus followed by the location of the locus in megabases (Mb) as denoted in the ARS-UCD 1.2 reference genome assembly (https://www.animalgenome.org/repository/cattle/UMC_bovine_coordinates/; accessed 5 September 2023). ^b^ Cow conception rate to first service (CCR1); Number of breeding services required to achieve conception (TBRD). ^c^ Inheritance model: additive (Add), dominant (Dom), recessive (Rec). ^d^ Genomic feature that the locus/SNP are located within: gene, regulatory regions, copy number variants (CNVs), conserved regions. *FDR < 0.05

The significant GBS SNP were then evaluated for LD with the original SNP that were used to create the custom assay. When compared to the original nine SNP used in to create the custom assay, the two GBS SNP in the locus on BTA1 at 84 Mb were in LD with only two of the original SNP (D’ > 0.75). The other significant GBS SNP on BTA1 at 146 Mb was in LD with one of the two original SNPs (D’ = 0.80), while the GBS SNP on BTA20 at 46 Mb was in high LD with both of the original SNP used to create the custom assay. The high level of LD with the original markers for the loci on BTA1 @146 Mb and BTA20 at 46 Mb suggests that progress can be made using the SNP on the currently available BeadChip. However, it should be noted that the two GBS SNP from the locus on BTA1 @84 Mb are only in LD with two of the nine original SNP indicting that the new GBS markers would be more useful for selection of fertility in cattle.

Loci associated with fertility in this study were compared to loci reported by Kiser et al. (2019a) [13] using only SNPs on the Illumina BovineHD BeadChip to determine if the GBS markers were helpful in further defining the validated CCR1 and TBRD loci. None of the three loci from the current studies were associated with fertility in Kiser et al. 2019a [13], meaning they were associated with cow fertility for the first time within the current analyses and could provide functional targets for further analysis to identify putative causal mutations. Originally, these loci were identified in two or more of the external fertility analyses investigated [15, 20, 30, 42] and associated with heifer fertility instead of cow fertility.

## Discussion

Most of the enriched gene sets have functions that fit within the organization of cellular components. These gene sets contain genes that are expressed within macromolecular complexes of the cell [57]. Gene Ontology’s cellular component designation characterizes genes whose molecular function occurs within a cellular compartment of the cell such as the plasma membrane or the cytoskeleton. The three most identified leading edge genes for CCR1 and TBRD (villin 1 (*VIL1)*, *RAC1*, and *TWF1*) were present within the gene sets in the organization of cellular components as they control different aspects of the cytoskeleton [58–60]. *VIL1* is often expressed on cell borders to help maintain their shape and their adhesion with other cells [61]. and its expression is also used as a marker for cells that have undergone the epithelial-to-mesenchymal transition, which is important for normal female reproductive organ function [62]. Similarly, *RAC1* is involved with uterine cell remodeling. A deficiency of *RAC1* expression in mice during the peri-implantation period impairs the function of the luminal epithelia reducing uterine receptivity [63]. Additional, several cattle studies have identified associations between several p21 activated kinases genes, which are regulated by *RAC1*, and fertility traits such as conception rate and pregnancy rate in Holsteins [25, 64]. Twinfilin Actin Binding Protein 1 is a highly conserved actin binding protein that regulates the assembly of the cytoskeleton through the binding of actin monomers [65]. While its function in cattle fertility is not characterized, in mice *TWF1* was the most common isoform of twinfilin in embryos as well as in non-muscle cell types within adults [65]. In general, the cytoskeleton plays an important role in all cells and is considered to have three overarching functions: (1) organizing the contents within a cell, (2) enabling the cell to move and change shape, and (3) connecting cells to their external environments [66]. 

Prior to blastocyst implantation, changes to the actin cytoskeleton of uterine epithelial cells can result in a loss of cell polarity [67, 68]. The loss of cell polarity can result in actin cytoskeleton disorganization leading to abnormal endometrial receptivity and implantation failure. In women, this failure influences the plasma membrane transformation period during the mid-secretory phase of the menstrual cycle [69]. In mice, cytoskeleton disorganization negatively impacts embryo development prior to implantation [70]. 

The second largest functional category of enriched gene sets is related to mitosis and the cell cycle. Mitotic cell division is an essential process for sustaining life. In the uterus, successful regulation of mitosis is important for fertility for the developing uterus, placenta, and embryo. Dysregulation of mitotic cell division leads to defects in uterine receptivity through alterations to stromal proliferation [71, 72] which could play a role in recurrent implantation failure in women [73]. During mitosis, centrosomes play a crucial role in the formation of the spindle assembly [74]. During reproduction, centrosomes are susceptible to rearrangements and changes to their complex structure can result in aneuploidy, mis-segregation of chromosomes, and destabilization of chromosomal integrity [75, 76]. Issues with centrosomes and their components can also result in oocyte abnormalities. Wang et al. (2001) [76] found that a main indicator of oocyte quality and thus chances of successful fertilization was centrosome integrity and its impact on spindle integrity.

Three gene sets (Neurotrophin Signaling Pathway, Notch Signaling Pathway, and Signaling by Neurotrophin Growth Factors [NGF]) in the signaling pathway category are involved in cellular proliferation and differentiation. Previous studies have found that NGF can increase cell proliferation through Notch signaling [77], and that the inhibition of Notch Signaling can result in the downregulation of NGF during acute inflammatory responses [78]. In addition to their nervous system roles, NGF and Notch Signaling function in ovulation [79] and pregnancy establishment [80], respectively. After systemic injection with NGF, luteinizing hormone and progesterone increase to aid in ovulation as well as establish and maintain pregnancy in llamas [79] and dairy cattle [81]. This increased expression of fertility hormones is suggested to increase uterine receptivity and reproductive efficiency. Notch Signaling has a broad role in female fertility from prenatal development, the estrus cycle, implantation and pregnancy [80]. Within the female reproductive tract, Notch Signaling activation is influenced by progesterone levels and has a role in pre-implantation angiogenesis by influencing the vasculature of the endothelial cells [80, 82]. Additionally, inhibition of Notch Signaling within the endometrium has been linked to repeated implantation failure, endometriosis, and polycystic ovary syndrome which cause infertility in women [83]. 

Gene sets enriched for CCR1 and TBRD that are related to RNA and mRNA (Table 4) could have several different influences on uterine receptivity and pregnancy maintenance. For example, some RNA and mRNAs are known to influence uterine receptivity during implantation through the regulation of Wnt signaling [84]. In humans, a meta-analysis on genes that were differentially expressed during embryo implantation and/or the endometrium identified 39 mRNA genes as uterine receptivity markers for uterine receptivity [85]. These genes have functions related to the immune response, exosomes, and the complement cascade pathway during successful pregnancies [85]. Alternative splicing of RNA and mRNA can also influence uterine receptivity. In mice, alternative splicing of epithelial splicing regulatory protein 1 (*Esrp1*) is associated with fertility, whereas *Esrp1*knockout female mice are infertile. Female Esrp1^−/−^ mice present with smaller ovaries and have impaired ovulation [86]. In humans, alternative splicing helps regulate gene expression within the myometrium to maintain quiescence throughout gestation [87]. Proteins involved in alternative splicing regulation like SF2/ASF and hnRNPA1 upregulate the production of certain GTP-binding protein isoforms during pregnancy leading to elevated cAMP levels and myometrial quiescence [88–89]. Alterations to RNA and mRNA processing and splicing have the potential to alter uterine receptivity and ultimately pregnancy success.

The association analyses identified 3 loci associated with CCR (*P* < 1 × 10^− 5^) from the 100 loci GBS panel. Two of the associated loci contained genes. Disco Interacting Protein 2 Homolog A (*DIP2A*) has potential functions in uterine receptivity while the function of *ENSBTAG00000032217* is currently uncharacterized. However, *ENSBTAG00000032217* is an ortholog to L-lactate dehydrogenase which is an enzyme that catalyzes the conversion of pyruvate to lactate as it converts NAD^+^ to NADH to produce energy in nearly all living cells. Previous work in mice has shown that *DIP2A* is expressed in the endometrium [90], and in bovine conceptuses during pregnancy recognition [91]. The DIP2A protein is also a receptor for follistatin which is essential for uterine receptivity [92, 93]. A successful pregnancy hinges on the ability of the embryo to implant into the endometrium. Further functional analyses are needed to determine how and if the variants identified in this study might influence uterine receptivity, implantation, and ultimately conception rate in dairy cows.

The observed heritability estimates from this study were moderate (CCR1 0.19 ± 0.05; TBRD 0.18 ± 0.05). When compared to the original heritability estimates calculated by Kiser et al. (2019a) [13] based on genome wide association analysis using 625,093 SNPs (0.56 ± 0.06 for CCR1 and 0.42 ± 0.07 for TBRD), the estimates from the current study were lower but similar to the heritability reported by others [94–97]. However, the current study heritability estimates confirm that significant improvement in fertility can be achieved by selecting for fertility traits. As one might expect, when the genetic correlation between CCR1 and TBRD was calculated it results in a genetic correlation of -1. This indicates that as the number of breedings needed to result in pregnancy increases, the conception rate to the first service decreases. Identifying variants and genes that are associated with both traits will help improve overall fertility within the industry.

The gene-sets, leading edge genes from the GSEA-SNP, the positional candidate genes, and the putative functional variants from the association study have potential roles in uterine receptivity and embryo implantation. Of the two loci that harbor positional candidate genes, one missense variant was located within the currently uncharacterized *ENSBTAG00000032217*, while two synonymous mutations were found within *DIP2A* and *ENSBTAG00000032217*. While the one missense variant is not thought to impact the function of the *ENSBTAG00000032217* protein, additional investigation is needed to confirm this due to the low confidence warning in the SIFT prediction.

Exploring putative causal mutations by genotyping through sequencing aided in defining the loci associated with fertility in dairy cattle. These results have practical implications in genomic selection as they could be incorporated into current commercial genotyping panels to facilitate genomic predicted transmitting abilities. This improvement in fertility would help alleviate financial losses dairy producers incur from failed pregnancies and increase their efficiency and profitability.

## Electronic supplementary material

Below is the link to the electronic supplementary material.

**Supplementary Material 1**: **Supplemental_Tables.xlsx**: Additional file 1 (.xlsx): **Table S1**. Milk yield data. **Table S2**. Gene sets associated with cow conception rate to the first breeding in Holstein cows. **Table S3**. Gene sets associated with number of breedings to conception in Holstein cows.

## Data Availability

Data generated and/or analysed during the current study are available in the CattleQTLdb repository (https://www.animalgenome.org/cgi-bin/QTLdb/BT/pubtails? PUBMED_ID=31718557) and the GBS data is pending review and acceptance by USDA Ag Data Commons (https://figshare.com/s/a748d0d75fbaf4303648).

## Abbreviations

AI: Artificial insemination
CCR: Cow conception rate
CCR1: Cow conception rate to first service
EMMAX: Efficient mixed-model association expedited
FDR: False discovery rate
GBLUP: Genomic best linear unbiased predictor
GBS: Genotype by sequencing
GSEA-SNP: Gene-set enrichment analysis with SNP data
LEG: Leading edge gene
NES: Normalized enrichment score
NGF: Neurotrophin growth factors
SNP: Single nucleotide polymorphism
SVS: SNP variation suite
TBRD: Number of times bred to conception
VCF: Variant call format
